# A novel Poly(ε-caprolactone)-Pluronic-Poly(ε-caprolactone) grafted Polyethyleneimine(PCFC-*g*-PEI), Part 1, synthesis, cytotoxicity, and *in vitro *transfection study

**DOI:** 10.1186/1472-6750-9-65

**Published:** 2009-07-17

**Authors:** Shuai Shi, QingFa Guo, Bing Kan, ShaoZhi Fu, XiuHong Wang, ChangYang Gong, HongXin Deng, Feng Luo, Xia Zhao, YuQuan Wei, ZhiYong Qian

**Affiliations:** 1 State Key Lab of Biotherapy and Cancer Center, West China Hospital, West China Medical School, Sichuan University, Chengdu, 610041, PR China

## Abstract

**Background:**

Polyethyleneimine (PEI), a cationic polymer, is one of the successful and widely used vectors for non-viral gene transfection *in vitro*. However, its *in vivo *application was greatly limited due to its high cytotoxicity and short duration of gene expression. To improve its biocompatibility and transfection efficiency, PEI has been modified with PEG, folic acid, and chloroquine in order to improve biocompatibility and enhance targeting.

**Results:**

Poly(ε-caprolactone)-Pluronic-Poly(ε-caprolactone) (PCFC) was synthesized by ring-opening polymerization, and PCFC-*g*-PEI was obtained by Michael addition reaction with GMA-PCFC-GMA and polyethyleneimine (PEI, 25 kD). The prepared PCFC-*g*-PEI was characterized by ^1^H-NMR, SEC-MALLS. Meanwhile, DNA condensation, DNase I protection, the particle size and zeta potential of PCFC-*g*-PEI/DNA complexes were also determined. According to the results of flow cytometry and MTT assay, the synthesized PCFC-*g*-PEI, with considerable transfection efficiency, had obviously lower cytotoxicity against 293 T and A549 cell lines compared with that of PEI 25 kD.

**Conclusion:**

The cytotoxicity and *in vitro *transfection study indicated that PCFC-*g*-PEI copolymer prepared in this paper was a novel gene delivery system with lower cytotoxicity and considerable transfection efficiency compared with commercial PEI (25 kD).

## Background

Gene therapy is being studied world-widely during the past 20 years. In almost all of experiments and clinical treatments, gene therapy requires delivering therapeutic gene into target cells to correct gene defects and achieve the purpose of treating diseases by using delivery carriers. People have never stopped pursuing more safe and efficient vector for gene delivery in gene therapy [1,2]. Gene delivery vectors can be generally divided into viral and non-viral vectors. Due to its high transfection efficiency, viruses were widely used before the potentially risk became serious [3]. The use of non-viral vectors may resolve some of the current problems associated with virus vector, such as safety risks. Due to lack of immunogenicity and improved transfection efficiency, non-viral vector is believed to be superior to viral gene delivery [4].

Non-viral vectors are ordinarily cationic that can condense negatively charged DNA into nano-complexes through electrostatic interaction. As a result, it can protect DNA from nuclease digestion, and thus enhance the expression of functional gene within the target cells. Therefore, various polycations were synthesized and have been investigated as gene carrier, including poly(L-lysine) [5], poly(aminoester) [6] and poly(propylene imine) (PPI) [7], polyethyleneimine [8-11], and etc. In all of the non-viral gene vectors, polyethyleneimine was regarded as a potential candidate with considerable transfection efficiency [8], but PEI has high cytotoxicity and short duration of gene expression [9-11]. Practically, transfection efficiency and cytotoxicity are almost antagonistic. PEI with low molecular weight (molecular weight = 800 Da, 2000 Da, or less) shows lower cytotoxicity and lower transfection efficiency, whereas PEI with high molecular weight (25 kD) shows higher transfection efficiency and higher cytotoxicity [12,13]. A novel gene delivery system needs to balance the transfection efficiency and cytotoxicity. Considerable attempts have been made to modify PEI in order to improve the biocompatibility, targeting and gene transfection efficiency [14-20]. Here we adopted the biodegradable and biocompatible PCFC to modify PEI to increase the transfection efficiency and decrease the cytotoxicity of PEI. Pluronic have been confirmed to increase the *in vitro *transfection efficiency and enhance the transgene expression *in vivo *[21,22]. Furthermore, due to grafting with Pluronic, biocompatibility of the polymer might be improved similar to the mechanism of PEGylation [23]. Zhao et al. [24] had reported cationic PCFC nanoparticles could condense DNA and have potential application as gene carrier with low cytotoxicity.

In this study, a novel poly(ε-caprolactone)-pluronic-poly(ε-caprolactone) grafted polyethyleneimine copolymer (PCFC-*g*-PEI) was synthesized and characterized. Meanwhile, the DNA condensation ability, protection ability, size and zeta potential of the PCFC-*g*-PEI/DNA complexes were detected. In addition, we observed considerable transfection efficiency and lower cytotoxicity of PCFC-*g*-PEI compared with PEI 25 kD.

## Results and discussion

### Synthesis of PCFC-g-PEI

**Figure 1:**
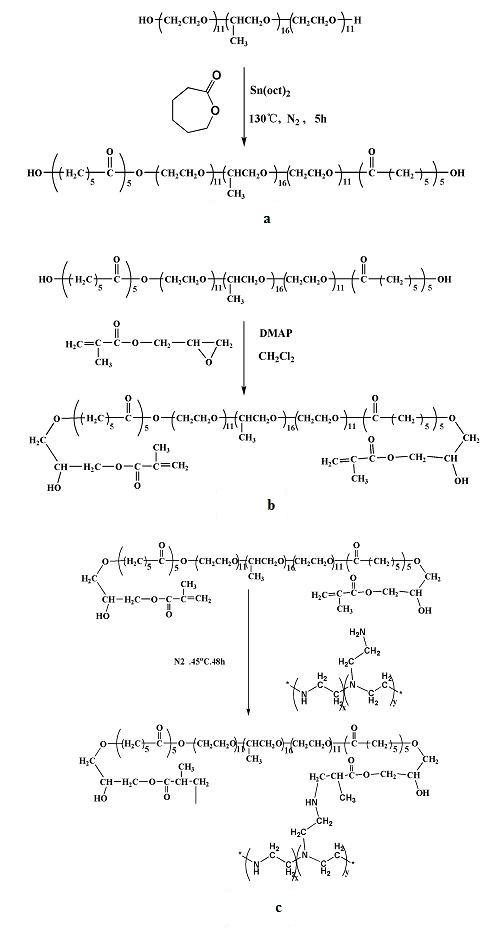
**a) Synthesis scheme of PCL-Pluronic-PCL (PCFC)**. b) Synthesis scheme of GMA- PCFC-GMA; c) Synthesis scheme of PCFC-*g*-PEI.

The aim of this study was to design and investigate an efficient non-viral gene carrier modified with PCFC. The PCFC-*g*-PEI copolymer was prepared according to Fig. 1 at three steps. At the first step of PCFC synthesis (Fig. 1-a), we chosen Pluronic 105 because it had satisfactory water-solubility; and the PCFC concentration in this copolymer had been optimized in prior research [24]. PCFC-*g*-PEI was prepared from PEI and PCFC, and the reaction scheme was shown in Fig. 1-c.

**Figure 2:**
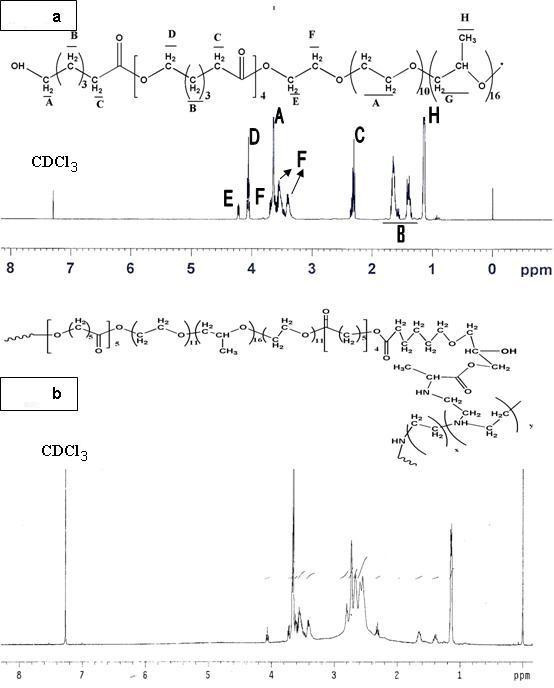
**^1^H-NMR spectrum (400 MHz) of PCL-Pluronic-PCL copolymer and PCFC-*g*-PEI in CDCl_3_**.

**Figure 3:**
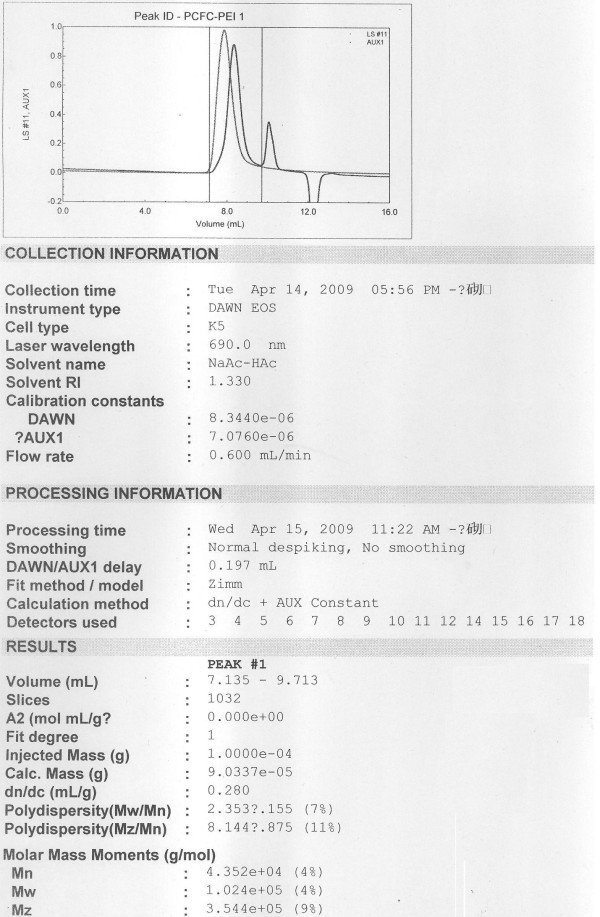
**Analysis of the PCFC-*g*-PEI by SEC-MALLS in 0.5 M sodium acetate-acetic acid**. The signals shown were detected by a refractive index detector.

The ^1^H-NMR spectrum of PCFC macromonomer was shown in Fig. 2-a. The peaks at 1.14, 3.42, and 3.50 ppm were attributed to protons of -CH_3_, -CHR-, and -CH_2_- in PPG unit of Pluronic block, respectively. The sharp peak at 3.65 ppm is attributed to methylene protons of -CH_2_CH_2_O- in PEG unit of Pluronic block. Peaks at 1.40, 1.65, 2.30 ppm, and 4.06 ppm are assigned to methylene protons of -(CH_2_)_3_-, -COCH_2_-, and -CH_2_OOC- in PCL blocks, respectively. The very weak peaks at 4.23 and 3.82 ppm are respectively attributed to methylene protons of -OCH_2_CH_2_- in PEG end unit linked with PCL blocks. The signals at 6.13 and 5.60 ppm corresponded to the protons of the double bonds and the signals at 1.4, 2.3, and 4.1 ppm corresponded to the protons of PCL segment respectively. The signal at 3.65 ppm corresponded to methylene proton of HOCH_2_- end group of PCL-GMA macromonomer. The ^1^H-NMR spectrum of PCFC-*g*-PEI macromonomer was shown in Fig. 2-b. Chemical shifts at 2–3 ppm of the ^1^H-NMR spectrums are attributed to the protons of -NHCH_2_CH_2_- of PEI. The chemical shifts at 3–4 ppm are attributed to the protons of Pluronic segment; the chemical shifts at 1–2 ppm and 4–5 ppm are attributed to the protons of PCL respectively. The molecular weight of the PCFC-*g*-PEI was determined by SEC in combination with multiple angle laser light scattering (MALLS) (Fig. 3). The absolute molecular weight was calculated by Astra software. The *M*_*n *_of PCFC-*g*-PEI was determined as 4.3 × 10^4^, whereas *M*_*w *_was 1.0 × 10^5^.

### Agarose gel electrophoresis

**Figure 4:**
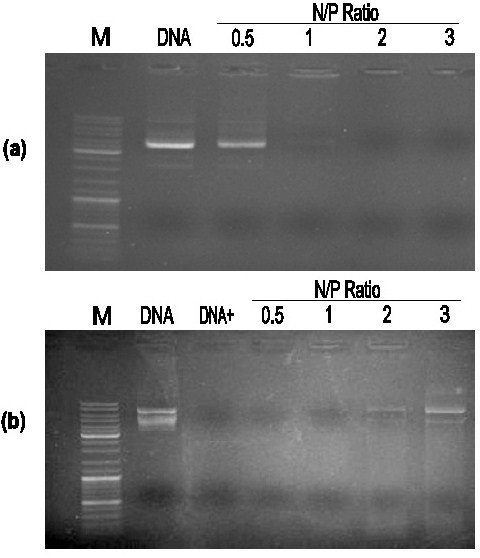
**(a) Agarose gel electrophoresis of PCFC-*g*-PEI/DNA complexes at various N/P ratios**. (b) Electrophoretic mobility analysis of PCFC-*g*-PEI/DNA complexes after DNase I treatment. DNA was released by adding 1 mg/ml heparin to the PCFC-*g*-PEI/DNA complexes. Both (a) and (b) used naked DNA as control.

The capacity to condense negatively charged DNA and protect DNA against DNase I degradation is necessary requirement for gene carriers. In this study, we characterized these abilities of PCFC-*g*-PEI by agarose gel electrophoresis. Fig. 4 showed agarose gel electrophoresis of PCFC-*g*-PEI/DNA complexes at various N/P ratios, and naked DNA was used as control. The N/P ratios of PCFC-*g*-PEI/DNA were 0.5, 1, 2, and 3, respectively, where the content of DNA was kept at 0.3 μg. As shown in Fig. 4-a, in contrast to the naked plasmid DNA, the migration of plasmid was completely blocked at the N/P ratio 1. In Fig. 4-b, the capability of protecting plasmid DNA from DNase degradation was examined using DNase I as model enzyme. Heparin was used as a strong negative reagent to destroy the PCFC-*g*-PEI/DNA complex and hence the plasmid DNA could be released. After incubated with DNase I (1 unit) in DNase/Mg^2+ ^digestion buffer at 37°C for 20 min, naked plasmid DNA showed significant degradation, while the plasmid DNA released from PCFC-*g*-PEI/DNA complexes (N/P = 3) remained intact. According to Fig. 4-a and Fig. 4-b, we can conclude that the PCFC-*g*-PEI copolymer can concentrate DNA at low N/P ratio and protect DNA from degradation by DNase-I.

### Size and zeta potential of the PCFC-g-PEI/DNA complexes

The size and zeta potential of the PCFC-*g*-PEI/DNA complexes are important factors for cell uptake [9]. Many factors could affect the results such as the concentration of DNA and polycations, the volumes of the complexes solution, the different sequences of addition of the reagents and also the speed of mixing. In this experiment, we chose deionized water as media when diluting and mixing PCFC-*g*-PEI with DNA, which was slightly different from some literatures [25]. According to Table 1 and Table 2, we found that the complexes of PCFC-*g*-PEI/DNA and PEI/DNA showed significant differences in size: when N/P ratio was below 10, the size of PCFC-*g*-PEI/DNA complexes was relatively stable around 200 nm, whereas the size of PEI/DNA complexes fluctuated from 90 nm to 160 nm. With increase in N/P ratio to 15 later, both of these complexes shown strong fluctuation. However, PCFC-*g*-PEI/DNA and PEI/DNA complexes showed the same trend in zeta potential change. With increase in content of PCFC-*g*-PEI, the zeta potential of PCFC-*g*-PEI/DNA complexes decreased until N/P ratio reached 10, and then it rebound to 7.8 mV. These results suggested that the DNA condensation ability was strongly increased at higher N/P ratio, In addition, we found that the zeta potential of these complexes was greatly affected by many factors such as mixing time, mixing sequence, and etc. All results in this part were measured three times and revised.

**Table 1:** The particle size of PCFC-*g*-PEI/DNA complexes, with PEI/DNA complexes as control. (SD = 3)

	Particle size (nm) (± SD)					
	
Samples	N/P = 1	N/P = 3	N/P = 5	N/P = 7	N/P = 10	N/P = 15
PCFC-g-PEI/pDNA complexes	208.05 ± 15.55	211.85 ± 11.95	215.3 ± 12.2	195.35 ± 6.95	161.4 ± 23.2	234.85 ± 10.25

PEI/pDNA complexes	118.5 ± 2.5	88.89 ± 0.83	99.115 ± 3.885	136.35 ± 4.45	159.1 ± 5.7	98.59 ± 9.81

**Table 2:** Zeta potential of PCFC-*g*-PEI/DNA complexes, with PEI/DNA complexes as control. (SD = 3)

	Zeta potential (mV) (± SD)					
	
Samples	N/P = 1	N/P = 3	N/P = 5	N/P = 7	N/P = 10	N/P = 15
PCFC-g-PEI/pDNA complexes	31.2 ± 2.1	16.75 ± 0.85	1.5125 ± 0.6575	1.38 ± 0.02	0.325 ± 0.19	7.835 ± 1.675

PEI/pDNA complexes	25.15 ± 0.75	21.25 ± 0.25	13 ± 0.7	14.8 ± 1.5	1.855 ± 0.235	0.651 ± 0.062

### Atomic force microscopy

**Figure 5:**
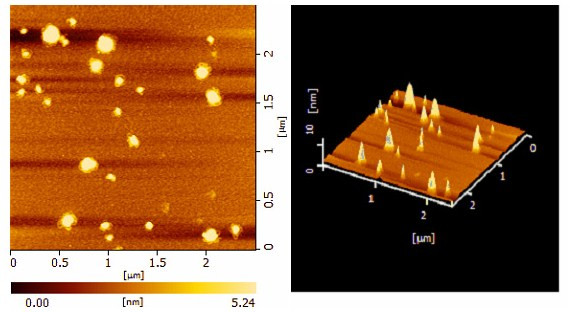
**Atomic force microscopy (AFM) image of the PCFC-*g*-PEI/DNA complex prepared at N/P ratio 7**.

The surface morphology of the PFCF-*g*-PEI/DNA complexes was determined by atomic force microscopy. As shown in Fig. 5, the complex of PCFC-*g*-PEI/DNA was distributed uniformly and the spherical nanoparticles were around 180 nm in diameter at N/P ratio 7.

### *In vitro *transfection

**Figure 6:**
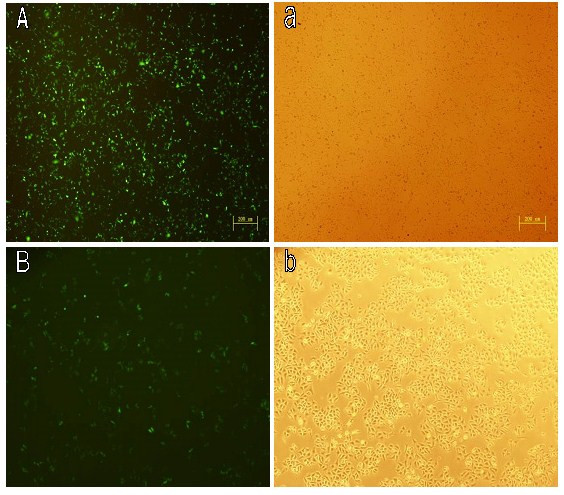
**Transfection images were shown in two cell lines**. A) 293 T; B) A549. Cells were incubated with PCFC-*g*-PEI/DNA complexes at N/P ratio 5 for 24 hours. The green fluorescent protein (GFP) expression was observed under fluorescent microscope at 5× magnification.

**Figure 7:**
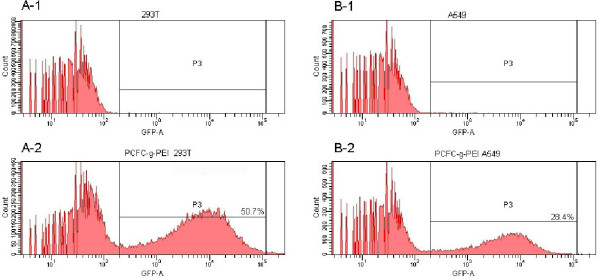
**The flow cytometry graphs of the transfection efficiency in two cell lines**. (A) 293 T and (B) A549. PCFC-*g*-PEI and PEI (25 KD) at optimized N/P ratios combine with DNA. A1 and B1 are the controls both used naked DNA, and A2 and B2 are the flow cytometry data that demonstrated the transfection efficiency.

The 293 T and A549 cells were seeded at 2 × 10^5 ^cells/well in 6-well plates and maintained at 37°C with 5% CO_2 _until the degrees of fusion reached 60%. Each experiment used the same protocol and the concentration of plasmid DNA was kept at 2 μg. After transient transfection and additional incubation for 24 h, transfection images were observed by Fluorescence Inverted Microscope (IX71, OLYMPUS), and photographed by using Spot Flex (Fig. 6), and transfection efficiency was quantitatively analyzed using flow cytometry (FCM). The highest transfection efficiency was observed in 293 T cell line which the efficiency reached 50.7%, while in A549 the transfection efficiency was 28.4% (Fig. 7).

Transfection efficiency depends on the charge ratio between PCFC-*g*-PEI and DNA. With optimizing N/P ratio, the transfection efficiency improved. In our work, the optimal N/P ratios of PCFC-*g*-PEI/DNA were 3 to 5, while that of PEI previously reported was 7 to 13[8]. The reason that PCFC-*g*-PEI/DNA had higher transfection efficiency at lower N/P ratio might be due to the high amine content because that PCFC-*g*-PEI was cascade connection using PEI 25 kD. There are less free elements when binding with DNA in transfection experiment so that the toxicity of PCFC-*g*-PEI will be reduced lower than that of PEI as the N/P ratio increased by observation [26]. In addition, as a Pluronic grafted copolymer, Pluronic 105 was contained in the PCFC complex and could be degraded in acid, it had been reported that Pluronic block copolymer exhibit valuable biological activities such as enhancing sealing of cell membranes permeated by ionizing radiation and electroporation thus preventing cellular necrosis [27,28], and could also enhance polycation-mediated gene transfer *in vitro *[29]. Due to these advantages, though the content of relative primary amine was decreased, the transfection efficiency did not decreased significantly according to our results.

### Cytotoxicity

**Figure 8:**
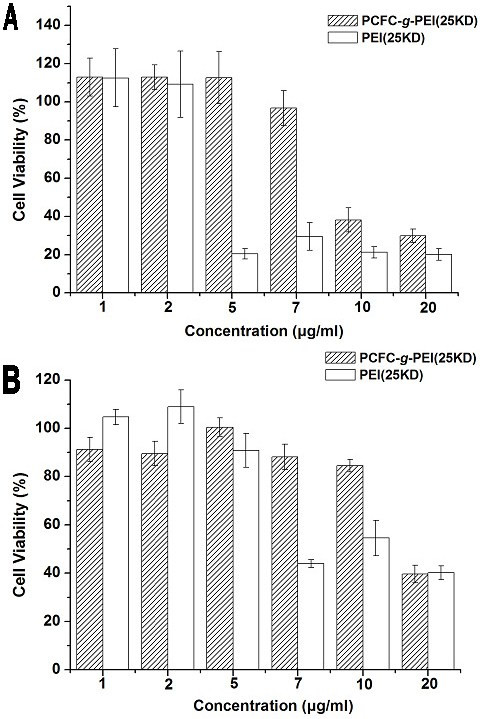
**Cytotoxicity of PCFC-*g*-PEI and PEI (25 KD) at various concentrations in two cell lines**. (A) 293 T and (B) A549. Cell viability were detected by MTT assay (mean ± SD, n = 3). The standard deviation is shown by the error bars.

**Figure 9:**
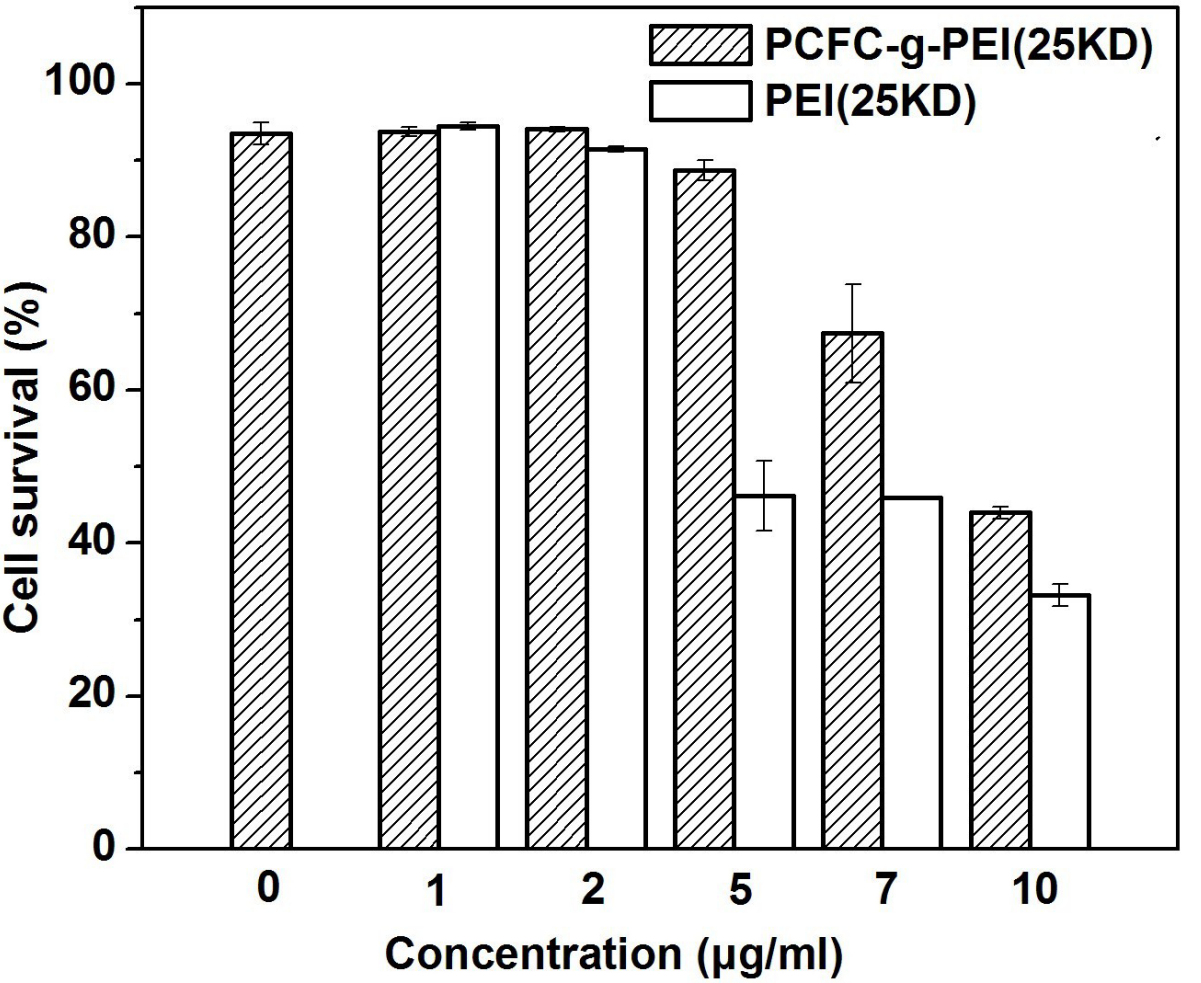
**The percentage of cell apoptosis caused by PCFC-*g*-PEI and PEI were studied through propidium iodide (PI) staining and flow cytometry (n = 3)**. Cell survival (%) = 1-apoptosis (%).

The cytotoxicity caused by PCFC-*g*-PEI (25 kD) and PEI (25 kD) were assessed by MTT assay (Fig. 8) and propidium iodide (PI) staining and flow cytometry (Fig. 9). MTT assay shows that complexes of PCFC-*g*-PEI have lower cytotoxicity than that of PEI 25 kD in two cell lines, 293 T and A549. It is confirmed that the cyto-compatibility of PCFC-*g*-PEI improved as compared with PEI 25 kD. In both cell lines, the tolerance of the gene delivery vector had increased to a different degree. The 293 T cell line was the most sensitive that demonstrated the biggest difference on tolerance between PCFC-*g*-PEI and PEI 25 kd. At the concentration of 5, 7 and 10 mg/ml, PCFC-*g*-PEI did not show apparent cytotoxicity, while the survival rate of PEI control group was approximately 40%. For the A549 cell line, both groups of PCFC-*g*-PEI and PEI showed higher tolerance than 293 T; and at the concentration of 10 μg/ml the cell viability of PCFC-*g*-PEI were greater than 80%, while the cell viability of PEI was just 70%. Many reports had confirmed that high molecular weight PEI with high gene transfection efficiency had higher significant cytotoxicity than low molecular weight PEI [30], but if complexes modified as PCFC, the cytotoxicity might decreased due to some reasons, such as the presence of biocompatible Pluronic [28], the decrease in free polymer concentration in cells [31], and the decrease in charge of complexes with decrease in primary amine amount.

The flow cytometry of cellular apoptosis caused by PCFC-*g*-PEI and PEI also indicated that PCFC-*g*-PEI have lower cytotoxicity than that of PEI 25 kD.

Of all the essays displayed, PCFC-*g*-PEI complexes could be used in gene delivery system with lower toxicity and considerable efficacy.

Many of reports had referred low molecular weight PEI been modified have various advantages and optimization of the reasons behind: size matters, molecular weight, zeta or degradable. There are very few studies on high molecule weight PEI modified or improved, due to the limitation in clinical application. However, as a comprehensive understanding of PEI transfer factor, the part of the high molecular weight PEI selected to conduct a feasibility study is necessary. And as a Pluronic block copolymer, the advantage of Pluronic may be used in improving the biocompatibility and enhancing the transfection efficiency in vitro. Following this study, we plan to regulate the molecular weight of PEI and the ratio between PCFC and PEI, and look forward to finding a series of cationic polymers as gene delivery system in gene therapy as soon as possible, and in vivo study of such PCFC-*g*-PEI copolymer will be studied in detail.

## Conclusion

In this work, we have synthesized a Pluronic block copolymer PCFC-*g*-PEI that crossed link with high weight molecular branched PEI (bPEI, 25 kD) by multiblock poly (ε-caprolactone)-Pluronic-poly (ε-caprolactone) (PCFC). The aim of this study was to increase transfection efficiency and cell permeability by grafting Pluronic with its biological activities. However, the transfection efficiency was not increased compared with bPEI 25 kD. PCFC-*g*-PEI effectively reduced toxicity on 293 T and A549, and retained considerable transfection efficiency *in vitro*. The size and zeta potential of PCFC-*g*-PEI/DNA complexes suggested that transfection efficiency depend on many factors such as the molecular weight of the polycation and the structure and biological activity agent. The Pluronic block of this cationic complex may be a very good reference on gene transferring both *in vitro *and *in vivo*.

## Methods

### Materials

Polyethyleneimine (PEI, Mw = 25 kDa), Pluronic 105 (poly (ethylene glycol)-poly (propylene glycol)-poly (ethylene glycol), PEG-PPG-PEG, Mw = 1900). ε-Caprolactone (ε-CL, Mw = 114), and glycidyl methacrylate (97%, GMA) were purchased from Aldrich (USA). Deoxyribonuclease I (DNase I), Dulbecco's Modified Eagle's Medium (DMEM) and 3-(4, 5-dimethylthiazol-2-yl)-2, 5-diphenyltetrazolium bromide (MTT) were bought from Sigma, USA. Dimethyl sulfoxide (DMSO), methanol, petroleum ether, and anhydrous dichloromethane were purchased from Chengdu KeLong Chemicals, China. All reagents and solvents were analytical pure grade.

### Synthesis of PCL-Pluronic-PCL (PCFC) copolymers

The PCL-Pluronic-PCL (PCFC) copolymers were prepared by ring-opening copolymerization of ε-CL initiated by Pluronic 105. PCFC copolymer was prepared by introducing calculated amount of ε-caprolactone and Pluronic 105 under nitrogen atmosphere into a dry three-necked bottle, and several drops of Sn(Oct)_2 _was added. The bottle was kept at 130°C. During polymerization, the system was stirred slowly, and the viscosity increased with time. After 5 hours, reaction was ended, and the reaction system was cooled to room temperature under nitrogen atmosphere. The just-obtained PCFC copolymer was first dissolved in methylene chloride and reprecipitated from the filtrate using excess cold petroleum ether. Then the mixture was filtered and vacuum dried to constant weight. The purified PCFC copolymer was kept in air-tight bags in desiccators before use. The reaction scheme was shown in Fig. 1-a.

### Synthesis of (GMA-PCFC-GMA)

The GMA-PCFC-GMA was obtained by reaction with PCFC and GMA. In this step, PCFC was dissolved in anhydrous dichloromethane and Glycidyl methacrylate (GMA) was also added according to introducing calculated amount. The reaction mixture was stirred for 48 h at 25°C. The obtained products were reprecipitated from the filtrate using excess cold petroleum ether for at least 4 times. The purified products were kept in air-tight bags in desiccators before use. The reaction scheme is shown in Fig 1-b.

### Synthesis of Branched PCFC-g-PEI

At last step of this synthesis, PCFC-*g*-PEI was synthesized by PEI grafted with GMA-PCFC-GMA by Michael addition of branched PEI (25 kD) to PCFC. The reaction scheme is shown in Fig. 1-c. Briefly, PEI and PCFC were separately dissolved in Methanol. The PCFC solution was slowly dropped into PEI solution by syringe with continuous stirring. The reaction mixtures were maintained at 45°C with nitrogen atmosphere and constant shaking for 48 h. Then the vials were cooled to room temperature and were vacuum-dried for 2 d. The obtained PCFC-*g*-PEI was dissolved in distilled water and dialyzed using bag filter (Mw = 50 kD) against distilled water for 72 h. After dialysis, the PCFC-*g*-PEI was lyophilized.

### Characterization of the macro monomer

The synthesized polymers PCL-Pluronic-PCL (PCFC), GMA-PCFC-GMA and PCFC-*g*-PEI were estimated by measuring Nuclear Magnetic Resonance (^1^H-NMR). Molecular weight of the PCFC-*g*-PEI copolymers were determined using SEC in combination with multiple angle laser light scattering (MALLS). Sodium acetate-acetic acid (0.5 M) was used as eluent with a flow rate of 0.6 ml/min. The refractive index detector from Merck-Hitachi (RI-71), equipped with an 18 angle light scattering detector (DAWN EOS, GaAs Laser 690 nm, 30 mW, K5 cell) from Wyatt Technology Corp. (Santa Barbara, CA) and in line with the RI detector, allowed the determination of the absolute molecular weight. The weight average MW was determined using a Zimm plot (ASTRA software version 4.90). Refractive index increments, dn/dc, for the PCFC-g-PEI copolymers were calculated with the same software using the data from the RI detector and the polymer concentration.

### Preparation of gene vector complexes

The whole coding sequence of green fluorescent protein (GFP) was inserted into pcDNA3.1 (Invitrogen, San Diego, CA) which contains a CMV promoter to construct the plasmid GFP expressing green fluorescent protein. And the pGFP solution was adjusted to 1 mg/ml. PCFC-*g*-PEI/DNA complexes were prepared at N/P ratios of 1, 3, 5, 7, 10, and 15. 2 μg of pGFP and the calculated amounts of polymers were each diluted in 100 μl of distilled water and gently mixed. The polyplex formulation was incubated at room temperature for 30 min before used.

### Determination of particle size and zeta potential of PCFC-*g*-PEI/DNA complexes

Particle size and zeta potential measurements of polymer/DNA complexes were evaluated using a Malvern Zetasizer 3000 HS (Malvern, UK). Polymer/DNA complexes were prepared in water at various N/P ratios. Before measurement, the complexes were incubated at room temperature for 10 min.

### Agarose gel electrophoresis

The agarose gel electrophoresis was used to determine capability of condensing negatively charged nucleonic acid and protection DNA from degradation. Plasmid DNA (pgfp-n1) was diluted to 0.1 mg/ml, cationic polymer solutions were then added to the plasmid solutions with the same volume at various [PEI]/[pDNA] N/P ratios and shortly vortexed. After 10 min incubation at room temperature, 10 μl complex solutions were analyzed by 1% agarose gel electrophoresis (90 V, 30 min). In the part of protection and release assay of DNA, PCFC-*g*-PEI/DNA complexes and naked DNA (0.3 μg) were separately incubated with DNase I (1 unit) in DNase/Mg^2+ ^digestion buffer consisting of 50 mM Tri-Cl, PH 7.6 and 10 mM MgCl_2 _at 37°C for 15 min. Then the complexes were treated with 4 μl of 250 mM EDTA for 15 min to inactivate DNase I and each sample was mixed with 2 μl of 1 mg/ml sodium heparin to release plasmid DNA. Finally, samples were incubated at room temperature for 2 h and run agarose gel electrophoresis using 1% agarose gel in TAE running buffer at 80 v for 40 min.

### Atomic force microscopy (AFM)

The morphology and size of the PCFC-*g*-PEI/DNA complexes were analyzed using atomic force microscopy (AFM). The complex solution was prepared by mixing 2 μg of plasmid DNA with polymer solution at N/P ratio 7. The complex solution was incubated at room temperature for 30 min and diluted in 1 ml distilled water, and then the complex solution was placed on mica surface and dried at temperature. Imaging was carried out using noncontact mode, and complexes sizes were determined by measuring the diameters of the complexes.

### *In vitro *transfection

For in vitro transfection, 293 T and A549 cells were cultured in DMEM medium containing Gln supplemented with 10% heated-inactivated fetal bovine serum and antibiotics (100 units/ml of penicillin, 100 units/ml of streptomycin). Cells were grown at 37°C in humidified air containing 5% CO_2 _and passage every 2–3 days. The cells (1 × 10^5 ^per well) were plated on 6-well plated for 24 h before transfection. Immediately before the initiation of transfection experiments, the medium was removed from each well and washed once with DMEM without serum and antibiotic and treated with fresh medium with a serial dilution of PCFC-*g*-PEI (25 kD). PEI/DNA complex was performed as control. Luciferase gene was monitored 24 h later by using Flow Cytometry (FCM).

### Evaluation of cytotoxicity

The cell cytotoxicity of the PCFC-*g*-PEI was determined by MTT analysis and propidium iodide (PI) staining and flow cytometry.

In the part of MTT assay, Cells were seeded in 96-well plates at a density of 1 × 10^4 ^(293 T) and 2 × 10^4 ^(A549) cells/well in 0.1 ml growth medium and incubated overnight, and then added 0.1 ml fresh DMEM growth medium containing a series of concentrations of PCFC-*g*-PEI to each well. Untreated cells in growth media were used as a control. Cell were incubated for 24 h and then followed by addition of 20 μl of 3-(4, 5-dimethylthiazol-2-yl)-2, 5-diphenyltetrazolium bromide (MTT) solution (5 mg/ml). After further incubation for 4 hours, the MTT solution (0.5 mg/ml) was carefully removed from each well, and 150 μl DMSO was added to dissolve the MTT formazan crystals. The absorbance was recorded at 570 nm by an ELISA microplate reader (Bio-Rad). The cell viability (%) was related to the control wells containing untreated cells with fresh cell culture medium and was calculated according to the following:



All dates are presented as the mean of there measurements (± SD).

In the part of propidium iodide (PI) staining and flow cytometry, cells were seeded in 6-well plates at a density of 2 × 10^5 ^(293 T) cells/well in 2 ml growth medium and incubated overnight at 37°C under 5% CO_2 _until the confluency reached 50~60%, and then changed the medium with 2 ml fresh DMEM growth medium containing various concentrations of PCFC-*g*-PEI to each well. Untreated cells in growth media were used as a control. Cell were incubated for 24 h and then collected for analysis. Survival (%) = 1 - apoptosis (%).

## Abbreviations

PEI: polyethyleneimine; PCFC: poly (ε-caprolactone)-pluronic- poly(ε-caprolactone); F105: poly (ethylene glycol)-poly (propylene glycol)-poly (ethylene glycol); GMA: glycidyl methacrylate 97%; DMEM: Dulbecco's Modified Eagle's Medium; MTT: 3-(4, 5-dimethylthiazol-2-yl)-2, 5-diphenyltetrazolium bromide; DMSO: dimethyl sulfoxide; MW: molecular weight.

## Competing interests

The authors declare that they have no competing interests.

## Authors' contributions

QZY, WYQ and ZX designed the experiments. And the research funds were supported by QZY and WYQ. QZY and LF corrected the manuscript. SS and GQF carried out experiments, analyzed the data, and wrote the manuscript; GQF and FSZ participated in the synthesis of the PCFC-g-PEI copolymer. GCY and DHX analyzed data and revised the manuscript. KB and WXH participated in the MTT cytotoxicity study.

All authors read and approved the final manuscript.
